# Benzalkonium Chloride Tolerance Among *Listeria innocua* from Food and Food Processing Environments in Poland

**DOI:** 10.3390/pathogens15010076

**Published:** 2026-01-10

**Authors:** Anna Zawiasa, Aleksandra Andrzejewska, Patryk Mikołajczak, Agnieszka Olejnik-Schmidt

**Affiliations:** Department of Food Biotechnology and Microbiology, Poznan University of Life Sciences, Wojska Polskiego 48, 60-627 Poznan, Poland; anna.zawiasa@up.poznan.pl (A.Z.); andrzejewska0720@gmail.com (A.A.); patrmik289@gmail.com (P.M.)

**Keywords:** *Listeria innocua*, benzalkonium chloride, food safety

## Abstract

Benzalkonium chloride (BC) is widely used as a disinfectant in the food industry; however, increasing reports of *Listeria innocua* isolates exhibiting tolerance to this compound highlight the need to better understand their adaptive mechanisms. This study aimed to evaluate BC tolerance in 51 *L. innocua* isolates originating from raw and processed meat products (*n* = 32) and meat-processing environments in Poland (*n* = 19). Phenotypic tolerance was assessed using the agar diffusion method on two media: Brain Heart Infusion (BHI) agar and Mueller–Hinton (M-H) agar supplemented with 1.2% sheep blood, across BC concentrations of 0, 5, 10, 15, 20, 25, 30, 35, 40, and 45 µg/mL, allowing the determination of minimum inhibitory concentrations (MICs). Genotypic analysis of tolerance determinants (*brcABC*, *ermC*, *qacE*, *qacF*, *qacG*, *qacH*, and *qacJ*) was performed by PCR. On BHI agar, MIC values ranged from 15 to 30 µg/mL, with 15 µg/mL most frequently observed, whereas on blood-supplemented M-H agar, MICs were lower (5–20 µg/mL), most commonly 10 µg/mL. Among tolerance-associated genes, *qacH* was the most prevalent (29% of isolates), followed by *brcABC* (4%) and *ermC* (2%), while the remaining genes were absent. These findings suggest that food products may serve as a reservoir for *L. innocua* isolates harboring tolerance to BC and contribute to a deeper understanding of how this species adapts to quaternary ammonium compounds.

## 1. Introduction

The genus *Listeria* comprises Gram-positive, facultatively anaerobic bacteria widely distributed in natural and food-processing environments [1]. Among them, *L. monocytogenes* is a well-recognized pathogen capable of causing listeriosis in both humans and animals [2,3,4]. In contrast, *L. innocua* is regarded as a non-pathogenic species and has long served as an indicator organism [5,6] and a surrogate model for *L. monocytogenes*, being extensively used in studies relevant to the food industry [7]. Its importance in the context of food safety is increasingly emphasized. *L. innocua* is the most frequently isolated *Listeria* species in food and food-processing environments [8], occurring either alone or together with other *Listeria* spp. Co-isolation alongside *L. monocytogenes* is reported to be the most common combination [7,9]. *L. innocua* is the species most frequently encountered in food-processing environments [10] and is particularly common in animal-derived foods, including poultry [11], beef [12], and ready-to-eat (RTE) meat products [13]. Beyond contaminated food, *L. innocua* is also commonly found within processing facilities, where it forms biofilms on both food-contact and non-contact surfaces [14]. Biofilm formation is a serious concern for the food industry because it enables bacteria to resist harmful environmental conditions [15]. *L. innocua* readily adheres to a variety of materials used in food-processing equipment, such as polymers, glass, polystyrene, and stainless steel [14,16]. Its ability to persist within multispecies biofilms, where it frequently co-occurs with *L. monocytogenes*, further enhances its survival and dissemination in processing environments. Biofilms in food-processing facilities are typically controlled through mechanical removal followed by the application of disinfectants [17].

Quaternary ammonium compounds (QACs) are widely used for the sanitation of both food-contact and non-contact surfaces [5,18,19]. The bactericidal activity of QACs results from their positively charged head groups binding to the negatively charged bacterial membrane, allowing hydrophobic side chains to disrupt membrane structure, cause cytoplasmic leakage, and ultimately lead to cell lysis [4]. Benzalkonium chloride (BC), a widely used QAC, is a common active ingredient in commercial disinfectants applied to sanitize surfaces in food-processing facilities [4,20,21] due to its high efficacy and its role in limiting the transmission of environmental pathogens [22]. However, bacteria in such settings are often exposed to sub-lethal disinfectant levels, which can create selective pressure leading to the acquisition of tolerance traits in initially susceptible strains. Indeed, *Listeria* spp. isolates from food-processing environments display a higher prevalence of QAC tolerance than isolates from human or general environmental sources [23,24]. Efflux pump systems represent a major mechanism contributing to BC tolerance [1]. Efflux systems reduce intracellular biocide accumulation, thereby enabling survival at elevated BC concentrations. These pumps may be encoded chromosomally or reside on mobile genetic elements. These include *qac* genes belonging to the small multidrug resistance (SMR) family [25,26], as well as Tn6188, a chromosomally integrated transposon that carries *qacH* and other related *qac* family determinants [27]. In addition, the *brcABC* operon, commonly located on pLM80-type plasmids [23,28] and encoding the TetR-family transcriptional regulator BcrA together with two SMR efflux pumps, BcrB and BcrC, has been widely associated with elevated BC tolerance [28]. The most recently described contributor is the *emr* efflux system, encoded on the genomic island LGI1 [29]. Additional efflux-related mechanisms have been described in *Listeria*, including the *mdrL* efflux pump [18,30,31] and *fepR*, a transcriptional regulator known to modulate efflux activity [19,32]. It has also been demonstrated that the VirAB-VirSR-AnrAB system, which comprises interconnected two-component signal-transduction pathways, contributes to increased BC resistance [31].

The importance of monitoring BC tolerance extends beyond disinfectant efficacy. Previous studies have demonstrated that QAC tolerance determinants frequently co-occur with genes conferring resistance to heavy metals such as cadmium and arsenic [22,33], raising concerns about co-selection in food-processing environments. Moreover, BC adaptation has frequently been shown to result in cross-adaptation to other antimicrobial agents [27,34] like clinically relevant antibiotics. Cross-resistance to chloramphenicol, clindamycin, penicillin, and streptomycin [34], as well as ciprofloxacin [32,35,36] and trimethoprim [37], has been documented. Such cross-resistance poses a potential threat to the effectiveness of antibiotic therapy for listeriosis [38].

Benzalkonium chloride (BC) is commonly applied on food-contact surfaces at concentrations of 150–400 mg/L. However, incomplete rinsing or improper application during sanitation may result in residual BC on surfaces at sub-use-level concentrations. Under such conditions, bacteria are exposed to low, sub-lethal amounts of the disinfectant. Because the concept of “resistance,” as defined for antibiotics, is not directly applicable to sanitizers, the term “tolerance” is increasingly used to describe reduced susceptibility at these sub-lethal concentrations. In this study, we therefore examined the ability of *L. innocua* isolates to grow in low concentrations of BC (<45 µg/mL).

The main aim of this study was to analyze the phenotypic tolerance of *L. innocua* isolates to BC and to investigate the occurrence of key genetic determinants associated with QAC resistance. In addition, the study sought to compare tolerance levels across different culture media and to assess potential relationships between MIC values and the presence of specific efflux-associated genes.

## 2. Materials and Methods

### 2.1. Bacterial Isolates

Isolates were obtained from the microbiological collection of the Department of Biotechnology and Food Microbiology at the University of Life Sciences in Poznan. Isolates were stored at −80 °C. All stored strains were resuscitated by streaking on Brain Heart Infusion (BHI, Oxoid, Warsaw, Poland) and incubation at 35 °C for 24 h. 51 *L. innocua* isolates (32 food isolates from raw meat products, including poultry, pork, and beef, as well as from processed meat products such as ham, sausage, and bacon, as well as 19 from the food processing environments obtained from October 2020 to November 2021) were used in the current study. Each of the isolates was previously identified phenotypically on ALOA agar (Agar Listeria Ottaviani and Agosti, Merck, Darmstadt, Germany). Additionally, all the strains were confirmed using two genetic techniques. Methodological details have been previously published [39].

### 2.2. Phenotypic Analysis

The sensitivity of the collected isolates to BC was assessed using an agar diffusion method. The antimicrobial agent was applied directly onto the agar surface as a drop of the tested solution, without the use of impregnated disks. Isolates preserved as glycerol stocks at −80 °C were used to inoculate BHI (Oxoid, Warsaw, Poland) agar plates. The inoculated plates were incubated at 35 °C for 24 h, and the obtained cultures were subsequently stored at 4 °C for up to three weeks for further use. A single colony from each culture was transferred into 100 µL of BHI broth and mixed thoroughly. A 5 µL drop of the resulting bacterial suspension was spotted onto BHI agar plates containing increasing concentrations of BC (0, 5, 10, 15, 20, 25, 30, 35, 40, and 45 µg/mL). Plates were then incubated at 30 °C for 48 h. The experiment was performed in three independent trials. Subsequently, the entire procedure was repeated using M-H agar (Muller-Hinton; Oxoid, Warsaw, Poland) plates supplemented with 1.2% defibrinated sheep blood (Oxoid, Warsaw, Poland), under identical conditions. The minimum inhibitory concentration (MIC) was defined as the lowest concentration of BC that completely prevented visible bacterial growth.

### 2.3. Genetic Analyses

Genetic analyses were performed with PCR using primers described in the literature. Genes associated with resistance and reduced sensitivity to QACs were analyzed (*brcABC*, *emrC*, *qacE*, *qacF*, *qacG*, *qacH*, *qacJ*). Primers used in the study were synthesized to order by Genomed S.A. (Warsaw, Poland). PCR reactions were performed in a T-Gradient thermocycler (Biometra, Göttingen, Germany) with the conditions given below in Table 1. Genes were analyzed in reactions using 0.2 U RUN polymerase (A&A Biotechnology, Gdańsk, Poland), with dedicated buffer, 0.2 mM nucleotide mix (A&A Biotechnology), and 0.5 µM of primers. Matrix DNA was added in the amount of 10 ng. Reactions were performed in 10 µL of final volume. PCR products were separated in 2–2.5% agarose gels containing Midori Green Advance (NIPPON Genetics EUROPE, Düren, Germany), at a final concentration of 1×. One randomly chosen sample for each gene was purified using the Clean-Up Concentrator kit (A&A Biotechnology) and sequenced by Genomed S.A. company. The sequences were then analyzed using the BLAST 2.15.0 tool (National Center for Biotechnology Information, Bethesda, MD, USA).

## 3. Results

### 3.1. The Prevalence of Tolerance to BC Among L. innocua Isolates

In this study, a total of 51 *L. innocua* isolates collected from meat products and meat processing environments were analyzed. The results of sensitivity testing of the isolates to BC are presented in Table 2. Phenotypic tolerance of *L. innocua* to BC has not been previously described in the literature. Therefore, in this study, we rely on the principles and methodological frameworks established for *L. monocytogenes.* Across studies on *L. monocytogenes*, the criteria used to define BC tolerance have not been consistent. One commonly used approach defines BC tolerance as an MIC exceeding 4 μg/mL [30]. Other studies, however, have applied higher thresholds, classifying strains as tolerant at MIC values of ≥8 μg/mL or ≥10 μg/mL [24,41]. More stringent criteria have also been proposed, in which strains with MICs ≥ 16 μg/mL, representing at least twice the MIC observed for most isolates (8 μg/mL), are considered BC-tolerant [26]. The absence of a standardized criterion for defining BC tolerance leads to substantial inconsistencies in how results are interpreted. In the present study, applying a threshold of >4 μg/mL [30] would classify all isolates as tolerant, whereas a threshold of ≥16 μg/mL would classify none [26]. Therefore, an intermediate threshold of ≥8 μg/mL was applied to enable a more informative interpretation of the results [24].

**Figure 1:**
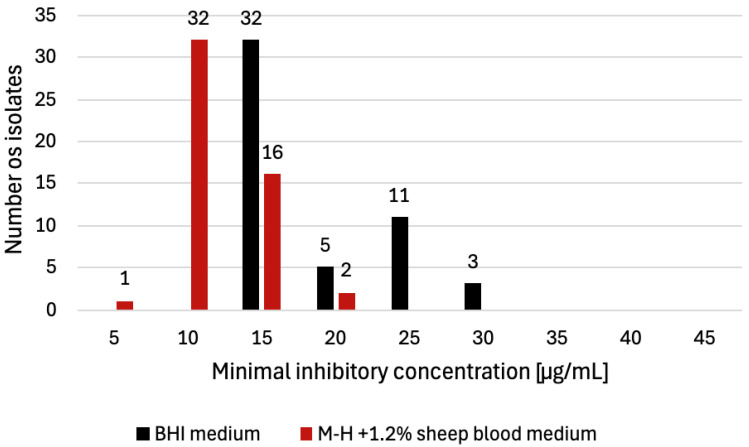
Distribution of *L. innocua* isolates (*n* = 51) according to benzalkonium chloride (BC) MIC values determined on two culture media. On BHI agar, the most frequently observed MIC was 15 µg/mL (*n* = 32), followed by 25 µg/mL (*n* = 11), 20 µg/mL (*n* = 5), and 30 µg/mL (*n* = 3). On M-H agar supplemented with 1.2% sheep blood, the most frequently observed MIC was 10 µg/mL (*n* = 32), followed by 15 µg/mL (*n* = 16), 20 µg/mL (*n* = 2), and 5 µg/mL (*n* = 1).

The assessment of phenotypic tolerance was conducted using two types of microbiological media: BHI agar and M-H agar supplemented with 1.2% sheep blood. MIC distributions obtained for each medium are shown in Figure 1. Because MIC values differ between media, the results are interpreted separately for each medium.

For BHI agar, the MIC values for all 51 isolates ranged from 15 to 30 µg/mL, with a mean value of 22.5 µg/mL. The most frequently observed MIC was 15 μg/mL (*n* = 32, 63%), followed by 25 μg/mL (*n* = 11, 22%), 20 μg/mL (*n* = 5, 10%), and 30 μg/mL (*n* = 3, 6%) (Figure 1). The highest MIC values (30 μg/mL) were observed in isolates originating from bacon (*n* = 1) and in isolates recovered from the production environment (*n* = 2). According to the ≥8 μg/mL criterion [24], all isolates tested on BHI agar were classified as BC-tolerant.

When evaluated on M-H agar supplemented with 1.2% sheep blood, the MIC patterns differed from those observed on BHI medium. The MICs for the tested isolates ranged from 5 to 20 µg/mL, with a mean value of 12.5 µg/mL. The most frequently recorded value was 10 μg/mL (*n* = 32, 63%), followed by 15 μg/mL (*n* = 6, 31%), 20 μg/mL (*n* = 2, 4%), and 5 μg/mL (*n* = 1, 2%) (Figure 1). The highest MIC values (20 μg/mL) were detected in the isolate originating from bacon (*n* = 1) and in the isolate recovered from the production environment (*n* = 1). Using a ≥8 μg/mL threshold [24], 50 of 51 isolates (98%) tested on M-H agar with sheep blood were classified as BC-tolerant. Overall, these MIC values were lower than those obtained on BHI agar. The MIC results for all isolates, with their sources and detected QAC determinants, are summarized in Table 2.

### 3.2. Prevalence of QAC Resistance Genes Among L. innocua Isolates

The analysis of genetic determinants of BC resistance included genes associated with reduced susceptibility to BC, specifically *qac*-family efflux pump genes (*qacE*, *qacF*, *qacG*, *qacH*, *qacJ*) as well as *brcABC* and *emrC*. PCR analysis of all isolates showed that *qacH* was the most frequently detected gene (*n* = 15, 29%), followed by *brcABC* (*n* = 2, 4%) and *emrC* (*n* = 1, 2%). None of the other investigated genes were detected. The gene presence results are summarized in Table 2. The isolates carrying resistance genes from the *qac, brcABC*, and *emrC* families originated from four different food and production-environment sources. The *emrC* gene was detected in an isolate obtained from the production environment, whereas *brcABC* was identified in two isolates recovered from the same food type (tenderloin). The *qacH* determinant was found in isolates from the production environment (*n* = 7), bacon (*n* = 6), and an additional sample from the meat-processing plant (*n* = 1).

A comparison of phenotypic and genotypic data revealed clear associations between MIC values and the presence of QAC resistance genes. On BHI agar, all isolates exhibiting the highest MIC value (30 µg/mL; *n* = 3) carried the *qacH* gene. Similarly, among isolates with an MIC of 25 µg/mL (*n* = 11), every isolate possessed at least one of the detected QAC-associated determinants (eight carried *qacH*, two carried *brcABC*, and one carried *emrC*). A comparable pattern was observed for results obtained on M-H agar supplemented with 1.2% sheep blood. All isolates with the highest MIC value of 20 µg/mL (*n* = 2) were *qacH*-positive, and among isolates with an MIC of 15 µg/mL (*n* = 16), eleven carried *qacH*, one carried *brcABC*, and one carried *emrC*. The remaining three isolates did not carry any of the examined resistance genes.

## 4. Discussion

In this study, two microbiological media—BHI agar and M-H agar supplemented with 1.2% defibrinated sheep blood—were used, and differences in the resulting MIC values were observed. To our knowledge, direct comparisons of BC MIC determinations obtained on these two media have not previously been reported for *Listeria* spp. The potential mechanisms discussed below therefore represent hypotheses rather than experimentally validated explanations. They are intended to provide plausible interpretations of the observed medium-dependent differences and to highlight directions for future experimental investigation. The differences in MIC values obtained on BHI agar and on M-H agar supplemented with 1.2% defibrinated sheep blood likely arise from a combination of chemical, physiological, and methodological factors. Variations in organic content, ionic composition, nutrient availability, and the physiological state of the bacteria may all influence the effective activity of BC. The higher concentration of organic compounds in BHI may bind or partially inactivate BC, reducing the fraction of freely active biocide and resulting in higher MICs. In contrast, M-H supplemented with sheep blood contains less organic material, potentially leaving a greater proportion of BC unbound, which may explain the lower MIC values observed. Differences in nutrient richness may also affect the metabolic state and stress-response capacity of the cells. Nutrient-rich BHI supports high metabolic activity, enabling bacteria to fuel energy-intensive defense mechanisms, including efflux pump activity and membrane repair, which can enhance survival in the presence of biocides. M-H with sheep blood is less nutrient-rich, limiting growth rate and cellular repair capacity, and thereby potentially contributing to the lower MIC values recorded on this medium. Additionally, the higher biomass formed on BHI may increase the amount of biocide required for complete inhibition, whereas the slower growth and lower biomass on M-H with sheep blood may require lower BC concentrations to suppress growth. Medium-dependent differences in membrane composition, ionic strength, and buffering capacity may further modulate susceptibility. The higher ionic strength and more complex ion composition of BHI may reduce the electrostatic attraction between positively charged BC molecules and the negatively charged bacterial surface. Stronger buffering capacity and the presence of divalent cations may also stabilize the cell membrane, decreasing its vulnerability to BC. Technical factors should also be considered. Components such as lipids, hemoglobin, and other organic molecules can influence optical clarity and color, affecting the visual interpretation of MIC endpoints. Different media may therefore alter the threshold at which growth appears inhibited, contributing to discrepancies in MIC values between BHI and MH with blood. Taken together, these hypotheses offer plausible explanations for the contrasting MIC values obtained on the two media. Results demonstrate that even moderate differences in medium composition can substantially influence MIC determinations, underscoring the importance of deliberate medium selection in biocide tolerance research. By addressing this methodological gap, this work highlights medium composition as a critical factor capable of modulating bacterial susceptibility assessments to disinfectants.

Under real processing conditions, during a standard sanitation cycle in the food industry, cleaning is performed prior to disinfection, and both cleaning agents and disinfectants are subsequently rinsed from surfaces. When rinsing is insufficient or disinfectants are applied improperly, residues of BC may remain on food-contact surfaces at concentrations below recommended use levels, thereby exposing bacteria to sub-lethal amounts of the compound [18]. In this context, the medium-dependent differences in BC MIC values observed in this study indicate that sanitizer performance under real processing conditions may vary depending on environmental factors, particularly the presence of organic matter. Higher MICs observed under nutrient-rich conditions suggest that residual organic loads on inadequately cleaned surfaces could reduce BC effectiveness. Together, these findings emphasize that sanitation programs should prioritize effective cleaning prior to disinfection and verify sanitizer performance under conditions that closely reflect actual processing environments rather than relying solely on standardized laboratory assumptions.

In terms of comparing results in this study with previous findings, one earlier investigation assessed the tolerance of 12 *L. innocua* isolates from environmental sources (swabs, water, and sewage) to various BC concentrations (0.25–12 µg/mL) on BHI medium, reporting MIC values between 1 and 6 µg/mL. Two isolates carried the *brcABC* gene, and although no additional resistance determinants were detected, the *brcABC*-positive isolates displayed higher MICs (4–6 µg/mL) than those lacking this gene (1–2 µg/mL) [19]. In this study, MIC values on BHI were substantially higher, ranging from 15 to 30 µg/mL. Similarly, two isolates carried the *brcABC* operon, and their MICs reached 25 µg/mL, which also exceeded the average MIC observed across all isolates. Although the MIC values reported previously were considerably lower, direct comparison remains difficult, as the earlier isolates originated from environmental settings with likely minimal exposure to BC, whereas the isolates examined here were recovered from food and food-processing environments, where contact with BC may plausibly occur. Another earlier study examined the tolerance of 75 *L. innocua* isolates, originating primarily from Polish meat products and the food-processing environment, collected between 2001 and 2010, using M-H agar supplemented with 1.2% sheep blood and BC concentrations of 0, 2.5, 5, 10, 20, and 40 µg/mL. In that work, isolates were classified as resistant if they showed confluent growth on agar containing 10 µg/mL BC (according to [41]), resulting in 9% of isolates (7/75) being considered BC-resistant [42]. When applying the same criterion to results obtained using the same medium but with isolates collected in 2020–2021, 50 of the 51 isolates (98%) would fall into the BC-tolerant category, with only one isolate (from the processing environment) classified as susceptible. While this proportion is higher than that reported in some previous studies, these differences should be interpreted with caution, as they may reflect variation in experimental conditions, applied MIC criteria, isolate origin, or collection sites rather than temporal changes. These findings underscore how differences in methodology and sampling context can substantially influence tolerance classification and highlight the need for standardized approaches when comparing data across studies. In study [8], 27 *L. innocua* isolates obtained from pork products were analyzed on M-H agar supplemented with 1.2% sheep blood using BC concentrations ranging from 0 to 30 µg/mL, with isolates classified as resistant when MIC values exceeded 10 µg/mL (criterion according to [41]). In that work, 14 isolates (52%) showed an MIC of 10 µg/mL (compared with 32 isolates, 63%, in this study), while nine isolates (33%) were classified as resistant with MIC values >10 µg/mL (compared with 18 isolates, 35%, in the current dataset). The highest MIC reported was 16 µg/mL (1 isolate, 4%), whereas in this study, the maximum MIC reached 20 µg/mL (1 isolate, 2%). In addition, the presence of QAC resistance determinants (*qacA/B*, *qacC/D*, *qacE*, *qacED1-sul*, *qacF*, *qacG*, *qacH*, *qacJ*, and *bcrABC*) was assessed by PCR. In contrast to these findings, where *qacH* (15 isolates, 29%) and *brcABC* (2 isolates, 4%) were detected, none of the isolates in that study carried any QAC resistance genes. A subsequent investigation conducted on *L. innocua* isolates from Poland further supports these observations. In that study, 50 isolates originating from meat products and food-processing environments were examined for the presence of the *brcABC* gene using PCR, and 6 isolates (12%) tested positive [42]. Together, these data indicate substantial variability in the distribution of QAC resistance genes among *L. innocua* populations, likely reflecting differences in ecological origin and exposure to disinfectants. This further suggests that no single genetic determinant can be considered universally responsible for the observed tolerance of isolates.

Importantly, in the analysis carried out here, the *emrC* gene was additionally detected in one isolate (2%) originating from the processing environment. To our knowledge, the presence of this genetic determinant has not been reported in *L. innocua* in the context of BC tolerance. Existing literature indicates that *emrC* occurs in *L. monocytogenes*, although with considerable variation in prevalence: 42% [43], 23% [5], 10% [44], and 6% [45]. The detection of *emrC* in *L. innocua* therefore expands current knowledge on the distribution of QAC-associated resistance determinants within the genus and indicates the potential for broader dissemination of this gene in food-processing environments. Such environments provide numerous opportunities for nonpathogenic *Listeria* spp. to coexist and interact with *L. monocytogenes.* Previous research has shown that BC resistance determinants such as *brcABC* can be transferred conjugatively from *L. innocua* and *L. welshimeri* to *L. monocytogenes*, as well as between nonpathogenic *Listeria* species themselves [10]. This supports the notion that nonpathogenic *Listeria* may act as reservoirs of resistance genes and facilitate their dissemination via mobile genetic elements both within their own populations and to *L. monocytogenes* occupying the same ecological niche.

The analysis demonstrated that the primary mechanism of tolerance or resistance to BC in *Listeria* involves the overexpression or functional modification of efflux pumps. All isolates exhibiting the highest MIC values on BHI agar (25–30 µg/mL; *n* = 14, 100%) carried at least one of the detected determinants (*qacH*, *brcABC*, or *emrC*). A similar trend was observed for M-H agar supplemented with sheep blood, where 15 of 18 isolates (83%) with the highest MIC values (15–20 µg/mL) also possessed one of these efflux-associated genes. The remaining three of eighteen isolates (17%) showing the highest MIC values on M-H with 1.2% sheep blood lacked all tested efflux pump genes, indicating that other mechanisms may contribute to reduced BC susceptibility. Such tolerance may arise through a combination of membrane-associated and regulatory adaptations. Proposed mechanisms include changes in membrane phospholipid and fatty acid composition, alterations in lipopolysaccharide structure, downregulation of porins, acquisition of transposon-associated elements, exposure to environmental stress factors, enhanced biofilm formation, and biodegradation of BC [20].

## 5. Conclusions

This study provides new insights into the phenotypic tolerance and genetic determinants associated with BC susceptibility in *L. innocua* originating from food and food-processing environments. The strong association observed between elevated MIC values and the presence of efflux-related genes, *qacH*, *brcABC*, and *emrC*, suggests that efflux-mediated mechanisms contribute to reduced BC susceptibility in this species. Future work should include functional validation of efflux activity, for example, using reserpine, an efflux pump inhibitor, which has previously been shown to reduce BC tolerance in *Listeria* by suppressing pump activity [8]. In addition, the high phenotypic tolerance observed in this study, with MIC values reaching up to 40 µg/mL, underscores the need for careful monitoring of BC use in food-processing environments. The pronounced medium-dependent variation in MIC values observed in this study further demonstrates that susceptibility assessments are strongly influenced by experimental conditions This includes ensuring that disinfectants are applied at appropriate concentrations, maintaining sufficient exposure times during sanitation procedures, thoroughly rinsing BC-based formulations to avoid residual sub-lethal concentrations, and eliminating crevices, recesses, or other hard-to-clean areas where disinfectant residues may accumulate and repeatedly expose *L. innocua* to low levels of biocides that promote tolerance development. Overall, the results obtained here enrich the global dataset on BC tolerance in *L. innocua* and contribute to a more comprehensive understanding of its distribution and potential underlying mechanisms. These findings highlight the importance of continuous surveillance, standardized interpretation, and proper disinfectant management to limit the emergence and spread of BC-tolerant strains in the food industry.

## Figures and Tables

**Table 1:** Detailed information about PCR conditions.

Gene Name	Primers Sequences	Cycling Condition	Primers [µL]	Amplicon Size [bp]	Reference
*bcrABC*	F: CATTAGAAGCAGTCGCAAAGCA R: GTTTTCGTGTCAGCAGATCTTTGA	94 °C 5 min; (94 °C 30 s; 57 °C 50 s; 72 °C 60 s) × 30; 72 °C 5 min	1.0	1100	[23]
*emrC*	F: TTATTCCATTTTATTACTGGCAATG R: CGTATTTATATTTAACACTAGCCA	94 °C 2 min; (94 °C 15 s; 50 °C 30 s; 72 °C 30 s) × 36; 72 °C 5 min	1.0	387	[40]
*qacH*	F: ATGTCATATCTATATTTAGC R: TCACTCTTCATTAATTGTAATAG	95 °C 5 min; (95 °C 25 s; 48 °C 40 s; 72 °C 40 s) × 35; 72 °C 5 min	1.0	366	[1]
*qacE*	F: AGCCCCATACCTACAAAG R: AGCTTGCCCCTTCCGC	94 °C 5 min; (94 °C 30 s; 55 °C 30 s; 72 °C 30 s) × 30; 72 °C 5 min	0.6	193	[26]
*qacF*	F: GTCATCGCAACTTCCGCACTG R: CTGACGATAAGTCCCAT	95 °C 5 min; (94 °C 30 s; 49 °C 30 s; 72 °C 45 s) × 35; 72 °C 5 min	0.5	245	
*qacG*	F: TAACTTACGCAACATGGGCA R: TCAATGGCTTTCTCCAAATAC	95 °C 5 min; (94 °C 30 s; 49 °C 30 s; 72 °C 45 s) × 35; 72 °C 5 min	0.5	156	
*qacJ*	F: CTTATATTTAGTAATAGCG R: GATCCAAAAACGTTAAGA	95 °C 5 min; (94 °C 30 s; 40 °C 30 s; 72 °C 45 s) × 35; 72 °C 5 min	0.5	306	

**Table 2:** The MIC results for isolates, with their origin and detected genetic determinants.

Isolate ID	Origin	Tolerance to BC		QAC Determinant						
		MIC BHI [μg/mL]	MIC M-H [μg/mL]	*brcABC*	*ermC*	*qacE*	*qacF*	*qacG*	*qacH*	*qacJ*
10	other	15	10	−	−	−	−	−	−	−
11	other	15	10	−	−	−	−	−	−	−
12	environment	15	10	−	−	−	−	−	−	−
13	other	20	10	−	−	−	−	−	+	−
14	poultry meat	20	15	−	−	−	−	−	−	−
16	poultry meat	15	10	−	−	−	−	−	−	−
32	environment	15	10	−	−	−	−	−	−	−
40	environment	15	10	−	−	−	−	−	−	−
41B	environment	15	10	−	−	−	−	−	−	−
46	sausage	15	10	−	−	−	−	−	−	−
54B	poultry meat	15	10	−	−	−	−	−	−	−
56	beef meat	15	10	−	−	−	−	−	−	−
57	beef meat	15	10	−	−	−	−	−	−	−
60	sausage	15	10	−	−	−	−	−	−	−
72	environment	15	10	−	−	−	−	−	−	−
80	environment	15	10	−	−	−	−	−	−	−
109	tenderloin	25	15	+	−	−	−	−	−	−
114B	environment	25	15	−	+	−	−	−	−	−
131A	environment	15	10	−	−	−	−	−	−	−
131B	environment	15	10	−	−	−	−	−	−	−
135	tenderloin	25	15	+	−	−	−	−	−	−
145	environment	15	10	−	−	−	−	−	−	−
146	bacon	20	15	−	−	−	−	−	+	−
150	bacon	25	15	−	−	−	−	−	+	−
158	environment	25	15	−	−	−	−	−	+	−
159	bacon	25	15	−	−	−	−	−	+	−
166	other	15	10	−	−	−	−	−	−	−
170	other	15	10	−	−	−	−	−	−	−
177	environment	15	10	−	−	−	−	−	−	−
187B	pork meat	15	10	−	−	−	−	−	−	−
193	ham	15	10	−	−	−	−	−	−	−
194	bacon	25	15	−	−	−	−	−	+	−
195	bacon	30	15	−	−	−	−	−	+	−
196	bacon	15	10	−	−	−	−	−	−	−
197	poultry meat	15	10	−	−	−	−	−	−	−
200	poultry meat	15	10	−	−	−	−	−	−	−
203	poultry meat	15	10	−	−	−	−	−	−	−
204	bacon	15	10	−	−	−	−	−	−	−
205	poultry meat	20	15	−	−	−	−	−	−	−
207B	poultry meat	15	10	−	−	−	−	−	−	−
214B	environment	15	10	−	−	−	−	−	−	−
215	sausage	15	10	−	−	−	−	−	−	−
228	environment	15	10	−	−	−	−	−	−	−
229	bacon	15	15	−	−	−	−	−	+	−
236	environment	20	5	−	−	−	−	−	+	−
238	environment	30	15	−	−	−	−	−	+	−
239	environment	30	15	−	−	−	−	−	+	−
240	environment	25	15	−	−	−	−	−	+	−
248	environment	25	15	−	−	−	−	−	+	−
249	environment	25	20	−	−	−	−	−	+	−
250	bacon	25	20	−	−	−	−	−	+	−

“−” indicates a negative result (gene absence), “+” indicates a positive result (gene presence).

## Data Availability

The original contributions presented in the study are included in the article; further inquiries can be directed to the corresponding authors.
